# Complement C1q stimulates the progression of hepatocellular tumor through the activation of discoidin domain receptor 1

**DOI:** 10.1038/s41598-018-23240-6

**Published:** 2018-03-20

**Authors:** Ji-Hyun Lee, Barun Poudel, Hyeon-Hui Ki, Sarmila Nepali, Young-Mi Lee, Jeon-Soo Shin, Dae-Ki Kim

**Affiliations:** 1 Department of Immunology and Institute for Medical Sciences, Chonbuk National University Medical School, Jeonju, Jeollabuk-do 54907 Republic of Korea; 2 Department of Oriental Pharmacy, College of Pharmacy and Wonkwang-Oriental Medicines Research Institute, Wonkwang University, Iksan, Jeollabuk-do 54538 Republic of Korea; 3 Department of Microbiology, BK21 PLUS for Medical Sciences, Yonsei University College of Medicine, Seoul, 03722 Republic of Korea

## Abstract

C1q is known to perform several functions in addition to the role it plays in complement activation. C1q contains a collagen-like portion and DDR1 (discoidin domain receptor 1) is a well-known collagen receptor. Accordingly, we hypothesized C1q might be a novel ligand of DDR1. This study shows for the first time C1q directly induces the activation and upregulation of DDR1, and that this leads to enhanced migration and invasion of HepG2 cells. In addition, C1q was found to induce the activations of mitogen-activated protein kinases (MAPKs) and phosphoinositide 3-kinase (PI3K)/Akt signaling, and to increase the expressions of matrix metalloproteinases (MMP2 and 9). Our results reveal a relationship between C1q and DDR1 and suggest C1q-induced DDR1 activation signaling may be involved in the progression of hepatocellular carcinoma.

## Introduction

Hepatocellular carcinoma (HCC) is one of the most aggressive liver tumors and accounts for ~5% of all malignant neoplasms. To date, no effective treatment strategy is available for HCC, and this would appear to be largely due to insufficient understanding of the etiologic and pathogenic mechanisms involved^1–3^. Therefore, it has been suggested improved understanding of the molecular targets underlying the development, metastasis, and invasiveness of hepatic cancer cells would certainly aid the developments of better treatments^4^. Many reports have described C1q biosynthesis in several different cells, such as, epithelial cells, fibroblasts, monocytes, dendritic cells, Kupffer cells, and microglial cells^5,6^. C1q plays an important role during tumor development, and a recent study reported that C1q contributes to C-activation and independent tumor growth, and that C1q can promote cancer cell adhesion, migration and proliferation^7^. Patients with HCC, acute hepatitis, chronic aggressive hepatitis, and patients with cirrhosis have elevated serum C1q levels, and it has been reported serum C1q levels increase with the progress of liver damage^8,9^.

C1 is the first component of the classical pathway of the complement (C) system and is composed of a complex of three proteins, that is, C1q, C1r and C1s. In serum, 70% of C1 is present as C1 complex and 30% is dissociated as C1q and C1r-C1s. C1q consists of three types of polypeptides chains (A, B, and C chains), each of which contain a C-terminal globular head region (gC1q) and a N-terminal collagen-like Gly/Pro-rich central repeat region (CLR)^10^. Normally, C1q binding to IgG molecules results in the classical pathway of complement activation^11^. However, C1q has many functions such as cell differentiation, migration, invasion, survival and adhesion, as well as its role as a complement that can be mediated by cell surface receptors^12,13^. To date, the receptors for C1q are known to the C1q spherical head region receptor such as gC1qR (p33 or C1qBP) and the collagen-like repeat region receptor such as calreticulin (CRT or cC1qR), CD93 (C1qRp), CR1, alpha2 beta 1 integrin, CD91 and LAIR-1^12,14^. Although several different cell types bind to and respond to C1q, the signaling mechanisms involved in the initiation of cellular response to C1q are not well understood^11^.

Disordin domain receptors (DDR) -1 and 2 are collagen receptors and members of a new class of receptor tyrosine kinases (RTKs) characterized by a 155-amino acid discordin homology domain in their extracellular regions. DDR consists of two types of non-integrin collagen receptors, DDR1 and DDR2, and is activated by receptor-specific collagen binding^15^. DDRs are activated by binding to collagen. DDR1 is activated by various types of collagen, including types I, II, III, IV, V, VI and VIII, whereas DDR2 is activated only by type I, II and III fibrillar collagens and non-fibrillar type X collagen^16–20^. However, DDRs are not activated by individual chains, denatured collagen, deglycosylated or degraded collagen^21,22^. The activations of collagen-mediated DDRs are characterized by slow, sustained activity^23,24^, and the up-regulation of DDR1 expression has been detected in several cancer types, including brain, esophageal, ovarian, and breast cancer^25,26^. Moreover, sustained DDR1 activation is known to regulate MMPs, which are well-known to be associated with the invasion and migration of cancer cells due to their abilities to degrade ECM components^27^. Previously, we reported that DDR1 overexpression enhances the migration and invasion of HCC cells via MMP production and thereby ECM degradation^26^. DDR1 is a well-known collagen receptor, but a report on collagen-independent signaling raised the exciting possibility that DDR1 has other as yet unidentified ligands^28^.

C1q has been reported to exhibit sequence and structural similarities with collagen VIII and X^29,30^ and it has been shown the collagen receptors α2β1 and LAIR-1, are also receptors for C1q collagen-like repeat regions^14,31^. So, we hypothesized that C1q might be a ligand of DDR1 (a known collagen receptor), and undertook this study, to investigate the molecular mechanisms of C1q and DDR1 in liver cancer cells.

## Results

### C1q enhanced the migration and invasion of HepG2 cells

**Figure 1:**
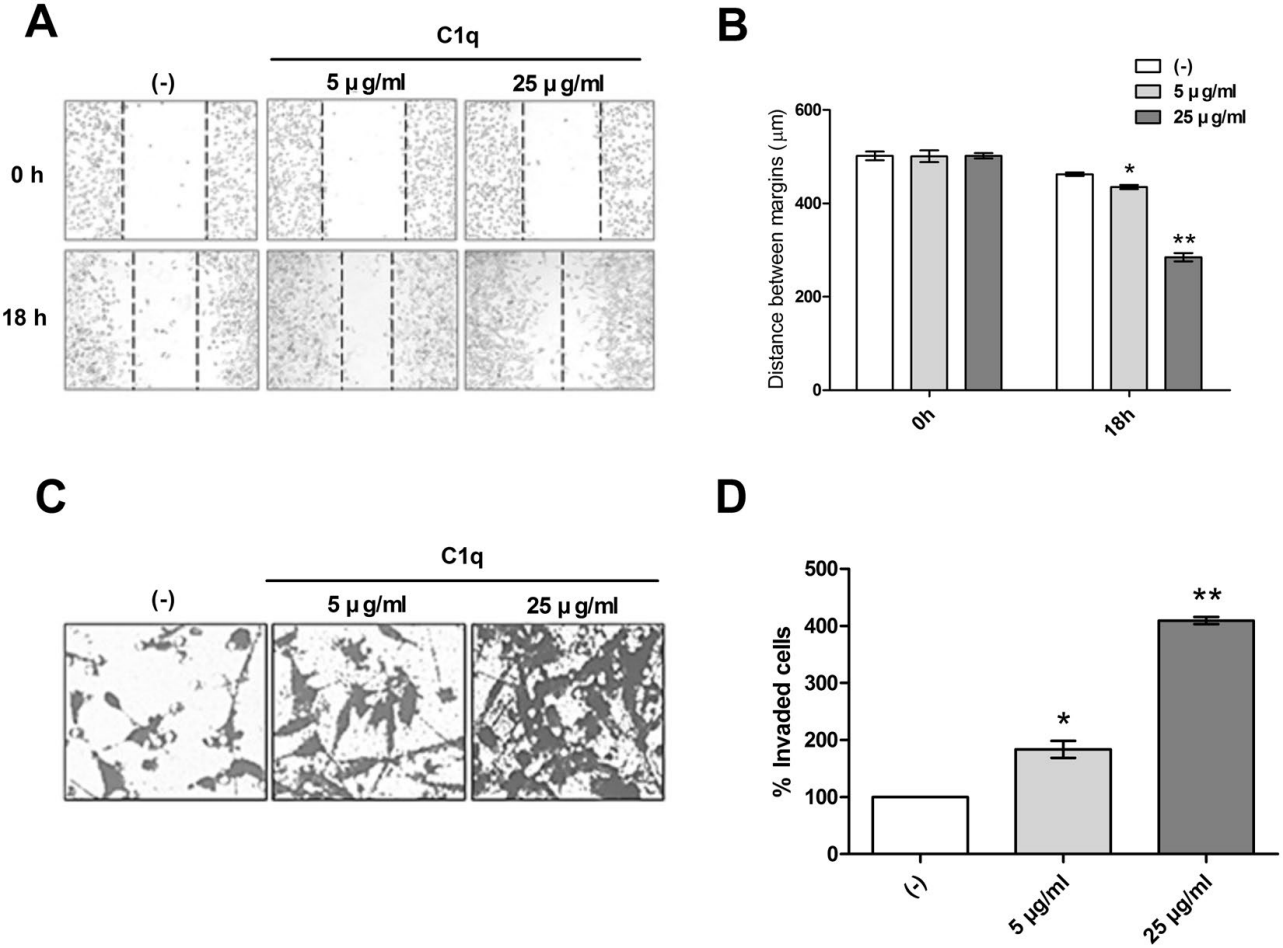
C1q enhanced the migration and invasion of HepG2 cells. A migration assay and a matrigel invasion assay were used to evaluate the impact of C1q on HepG2 cells migration and invasion. (**A**) Migration assays showed C1q increased the ability of HepG2 cells to migrate. The images were taken 0 and 18 h after monolayers were scratched. (**C**) Matrigel invasion assays showed that C1q enhanced HepG2 cell invasiveness. (**B**) Migrated distances and (**D**) numbers of invading cells were determined in at least 6 randomly selected microscope fields at 10 × and are shown in the bar diagram. *P < 0.05.

Many studies have shown C1q is involved in cell migration and invasion^32^. Thus, we examined the involvement of C1q in the migration and invasion of liver cancer cells. Migration and invasion assays showed average numbers of migrating and invading HepG2 cells were significantly increased by C1q (Fig. 1A–D).

### C1q enhanced MAPKs signaling in HepG2 cells

**Figure 2:**
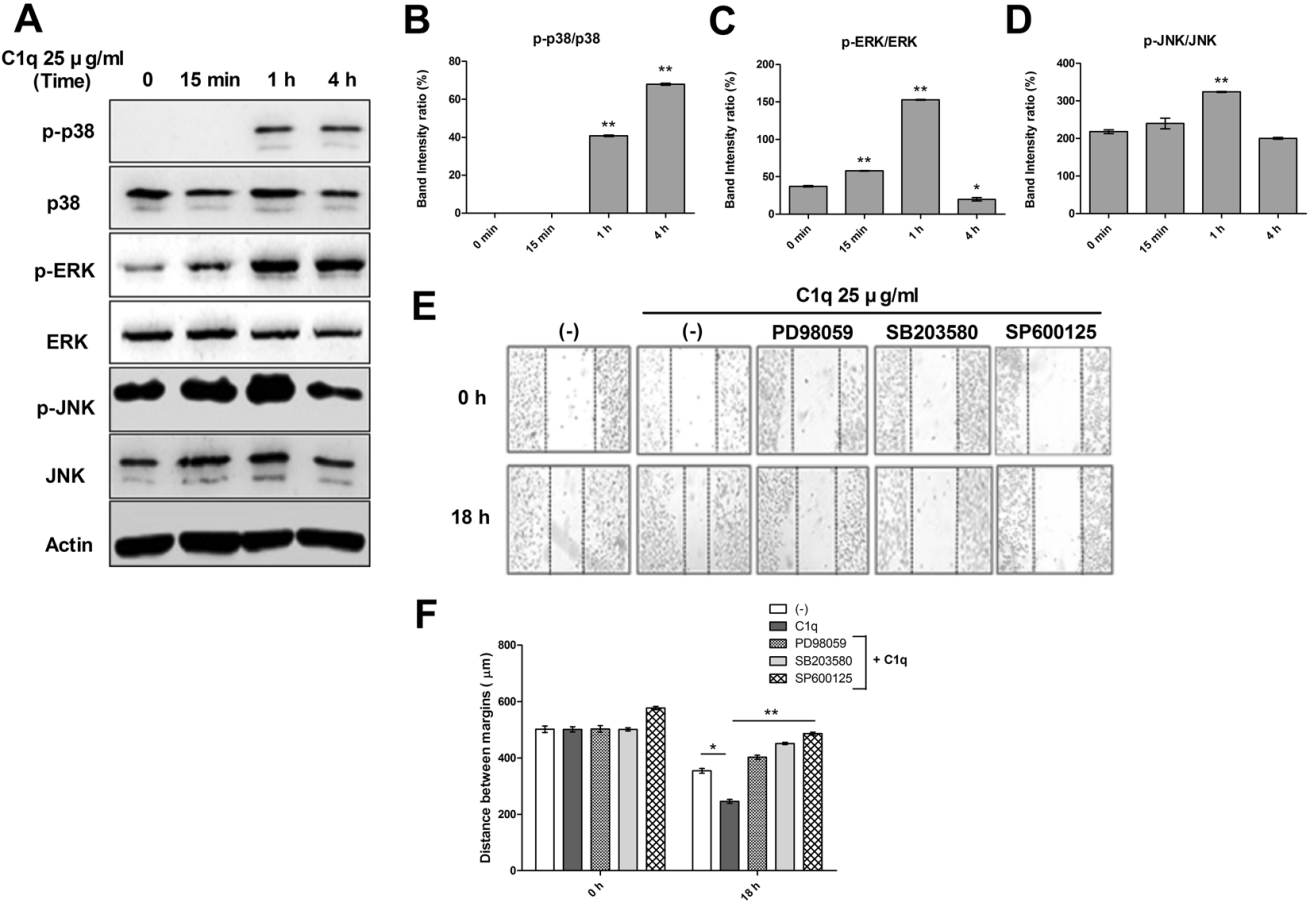
C1q enhanced MAPKs signaling in HepG2 cells. After treating HepG2 cells with or without C1q for the indicated times, the expressions of the phosphorylated and total forms of p38, ERK1/2, and JNK were evaluated by western blotting (**A**). Bar graphs represent the following ratios; p-p38/p38 (**B**), p-JNK/JNK (**C**), and p-ERK1/2/ERK1/2 (**D**). Cells were pretreated with MAPKs inhibitors (20 µM each) for 1 h, then monolayers were scratched, and then treated with or without C1q. Photographs were taken after treatment for 0 or 18 h (**E**). The bar graph shows migration distances (**F**). *P < 0.05, **P < 0.01.

To investigate the mechanisms responsible for the C1q-induced promotions of HepG2 cell migration and invasion, we examined levels of the MAPKs proteins p38, ERK, and JNK in cells treated with or without C1q. These proteins were selected because they are crucial for the induction of tumor cell epithelial-mesenchymal transition (EMT)^33^ As shown in Fig. 2A–D, levels of p38 MAPKs were significantly increased in HepG2 treated with C1q for 1 h, and levels of ERK1/2 and JNK were slightly increased at 1 h and returned to baseline at 4 h. To determine whether C1q causes cell migration through MAPK signaling, cells were pretreated with PD98059 (MEK1 activation and MAP kinase cascade inhibitor), SB203580 (p38 MAPK inhibitor), or SP600125 (JNK inhibitor), respectively, and then treated with C1q. Pharmacological blockade of MAPKs attenuated C1q-induced HepG2 migration (Fig. 2E and F), which suggested C1q might increase HepG2 migration by enhancing signaling through the MAPK pathway.

### C1q increased PI3K/Akt signaling in HepG2 cells

**Figure 3:**
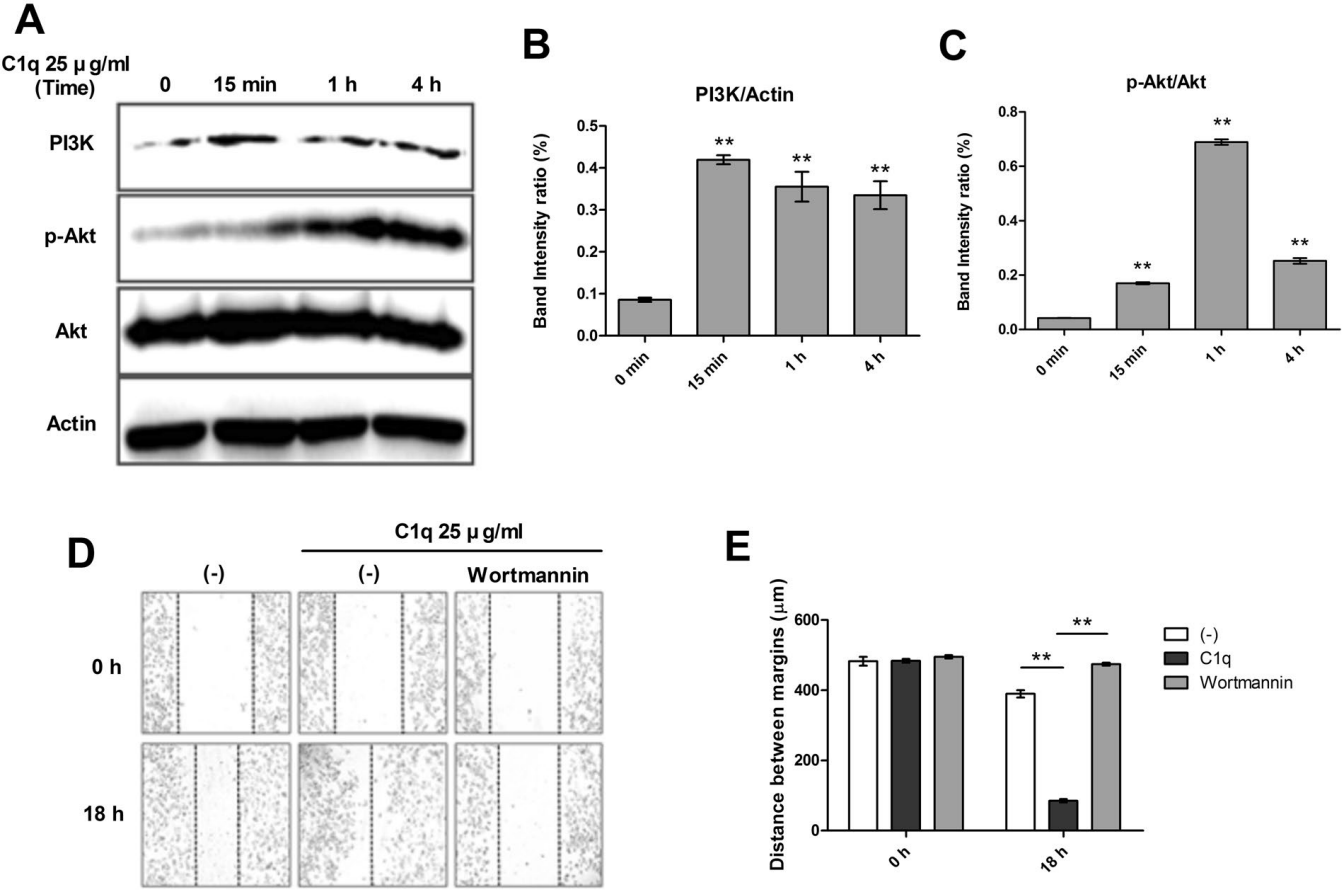
C1q activated the PI3K/Akt signal pathway in HepG2 cells. The effects of C1q on the expressions of PI3K and Akt in HepG2 cells were determined by western blotting. C1q enhanced the expressions of PI3K and of phosphorylated Akt. (**A**) HepG2 cells were treated with 25 µg/mL C1q for 15 min, 1 h, or 4 h. Bar graphs of (**B**) PI3K/Actin and (**C**) p-Akt/Akt ratios. (**D**) Pharmacological inhibition of the PI3K/Akt signal pathway by wortmannin (20 µM) pretreatment for 1 h inhibited migration significantly more in the presence of C1q than in the absence of C1q. (**E**) Bar diagram showing migration distances. *P < 0.05, **P < 0.01.

Activation of the PI3K/Akt pathway downstream of DDR1 signaling is reportedly involved in the development and progression of cancer^34,35^. To determine whether C1q augments PI3K/Akt signaling, HepG2 cells were treated with or without C1q and then subjected to Western blot analysis for PI3K, phosphor-Akt and total Akt. As shown in Fig. 3A–C, levels of PI3K and phosphor-Akt were significantly increased by C1q treatment. Consistent with activation of the PI3K/Akt pathway, we observed inhibition of C1q-induced migration upon pharmacological blockade of the PI3K/Akt pathway (Fig. 3D and E). Taken together, these findings suggest that C1q may enhance migration by augmenting PI3K/Akt signaling.

### DDR1 was activated by C1q in HepG2 cells

**Figure 4:**
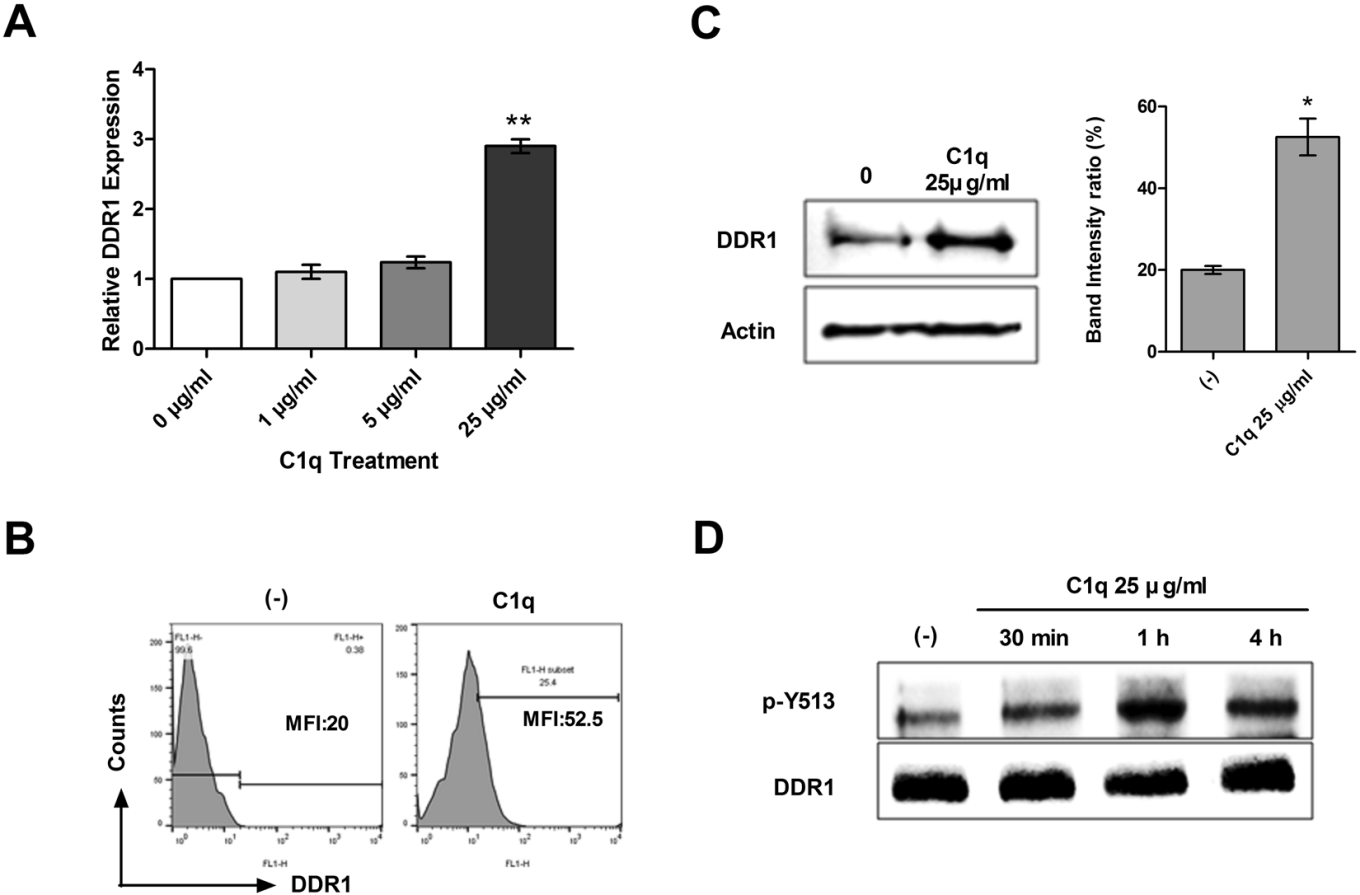
C1q targeted DDR1 in HepG2 cells. Cells treated with or without C1q were harvested and DDR1 mRNA level was assessed by real-time PCR (**A**). Cells were treated with the indicated doses of C1q for 18 h and DDR1 expressions were analyzed by flow cytometry (**B**) and Western blot (**C**). Levels of phosphor-DDR1 and total DDR1 in cells treated with C1q for the indicated times were assessed by immunoprecipitation followed by western blotting analysis (**D**). *P < 0.05, **P < 0.01.

C1q consists of C-terminal globular head region (gC1q) and N-terminal collagen-like repeat region (CLR)^10^. Here, we investigated the effect of C1q on gC1q receptors, such as, C1qBP (gC1qR), and on cC1q receptors, such as, calreticulin (cC1qR), CD93, α2β1 integrin, CD91, and LAIR-1, and its effects on DDRs. The up-regulation of DDR1 by C1q treatment was evaluated by real-time PCR, flow cytometry, and western blot. As shown in Fig. 4A–C, enhanced DDR1 expression was observed in C1q-treated HepG2 cells, but DDR2, CD91, β1 integrin, and LAIR1 were not detected. In addition, though C1qBP (gC1qR), calreticulin (cC1qR), and CD93 were expressed, these expressions were unaffected by C1q (see Supplementary Fig. S1). Additional experiments were performed using SUN182 cells (another HCC cell-line), and similar results were obtained (Supplementary Fig. S2). Levels of DDR1 phosphorylation were assessed by immunoprecipitation using DDR1 antibody (sc-532) and subsequent immunoblot analysis using phospho-DDR1 (Y513) antibody (P00905). As shown in Fig. 4D, the phosphorylation of DDR1 was significantly enhanced after 1 h of C1q treatment. To our knowledge, this is the first time that C1q has been reported to activate DDR1 directly in HepG2 cells.

### Knockdown of DDR1 suppressed the C1q-induced migration and invasion of HepG2 cells

**Figure 5:**
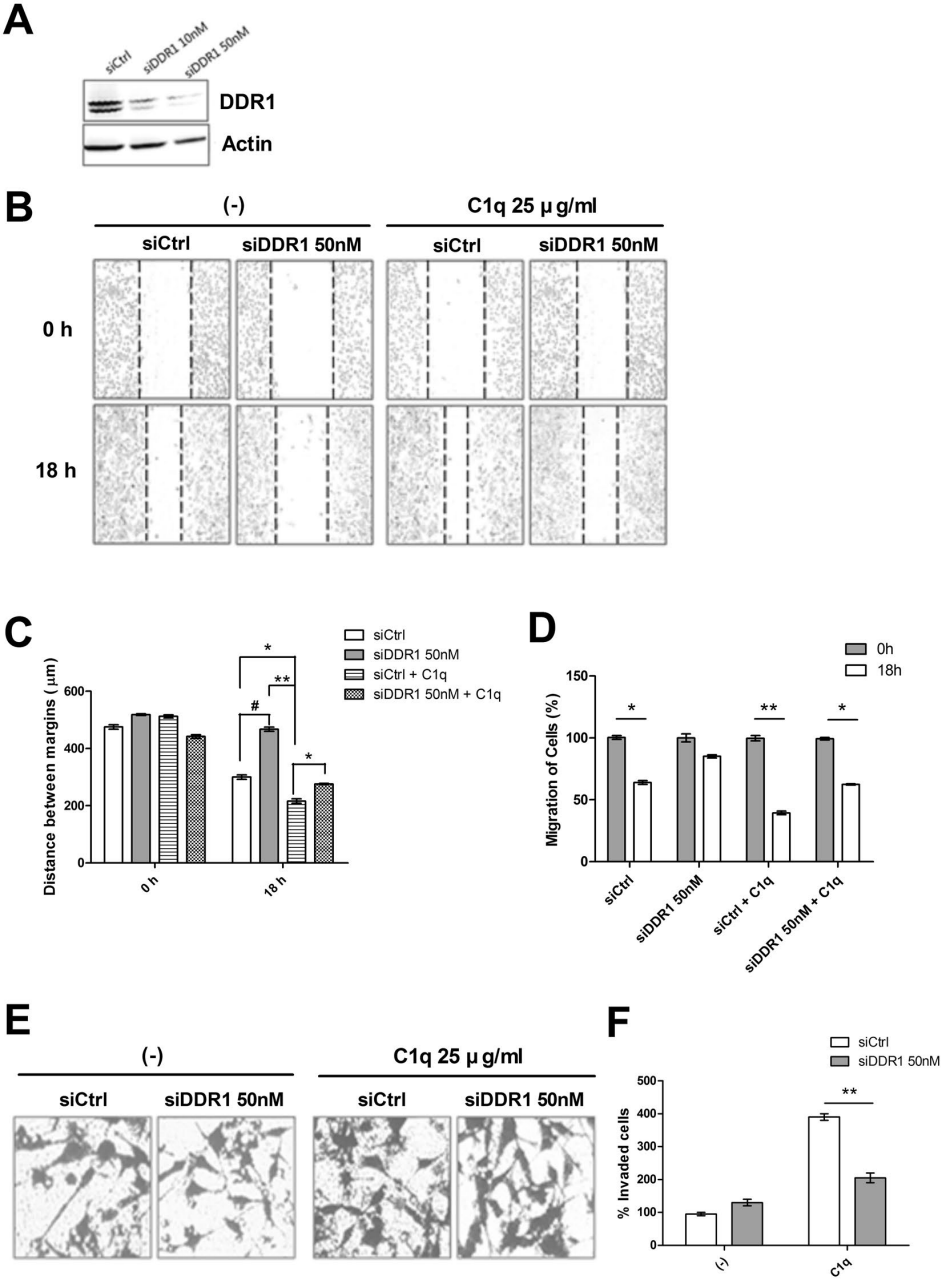
Knockdown of DDR1 attenuated the C1q-induced migration and invasion of HepG2 cells. Evaluation of DDR1 protein levels by western blotting after transfection for 36 h with scrambled (siCtrl) or DDR1-specific siRNAs (**A**). Control or DDR1 siRNA-transfected cells were cultured in 48-well plastic plates and allowed to adhere. A linear wound was then created in each well using a pipette and cells were treated with or without C1q to initiate migration (**B**). Transfected cells were cultured in transwell inserts in the presence or absence of C1q for 18 h and invading cells were counted as described in Materials and Methods (**E**). Bar diagrams representing migration distances (**C**), and counts of migrating cells (**D**) and invading cells (**F**). *P < 0.05, **P < *0.01* vs siCtrl + C1q, ^#^*P* < *0.05* vs siCtrl.

DDR1 signaling regulates the migration and invasion of tumor cells^27^, and thus, we examined the involvement of DDR1 in the C1q-mediated progression of hepatic cancer. In HepG2 cells DDR1 siRNA transfection dose-dependently reduced DDR1 expression (Fig. 5A). In the presence of C1q, the migration and invasion of DDR1 siRNA-transfected cells were inhibited as compared to that observed in control siRNA-transfected cells (Fig. 5B–F).

### C1q induced the expressions of MMP2 and 9 in HepG2 cells

**Figure 6:**
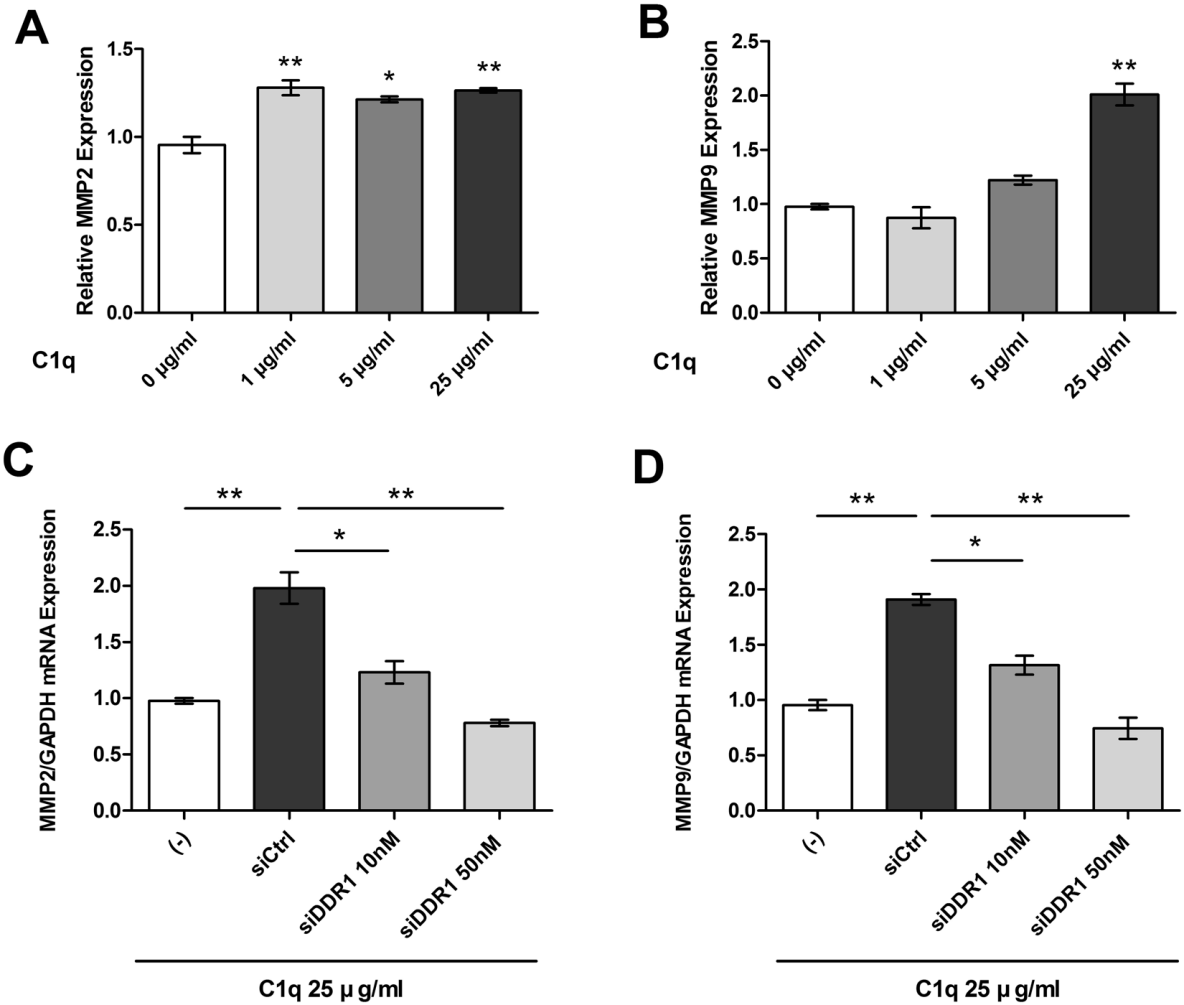
C1q induced the expressions of MMPs in HepG2 cells. The effects of C1q on the expressions of MMP2 and MMP9 in HepG2 cells were evaluated by real-time PCR. (**A**) Real-time PCR for MMP2 in C1q treated HepG2 cells. Cells were treated with or without the indicated doses of C1q for 6 h, harvested, total RNA was prepared, and reverse transcription reactions were performed using SYBR Select Master Mix Reagent. Real-time PCR was then used to assess MMP2 expressions. (**B**) Real-time PCR for MMP9 in C1q treated HepG2 cells. Cells were transfected with control (siCtrl) or DDR1-specific siRNA for 36 h and then treated with or without C1q for 6 h. The mRNA expressions of MMP2 (**C**) and MMP9 (**D**) were determined by real-time PCR. *P < 0.05, **P < 0.01.

C1q treatment significantly enhanced the expressions of MMP2 and 9 (Fig. 6A and B), which suggested C1q might augment cancer progression by up-regulating these MMPs. Furthermore, C1q-induced expressions of MMP2 and 9 were significantly reduced by DDR1 knockdown (Fig. 6C and D). These findings suggest that C1q-mediated activation of DDR1 and upregulations of MMP2 and 9 are required for the metastasis and invasion of HepG2 cells.

### C1q regulated epithelial-mesenchymal transition (EMT) via DDR1 in HepG2 cells

**Figure 7:**
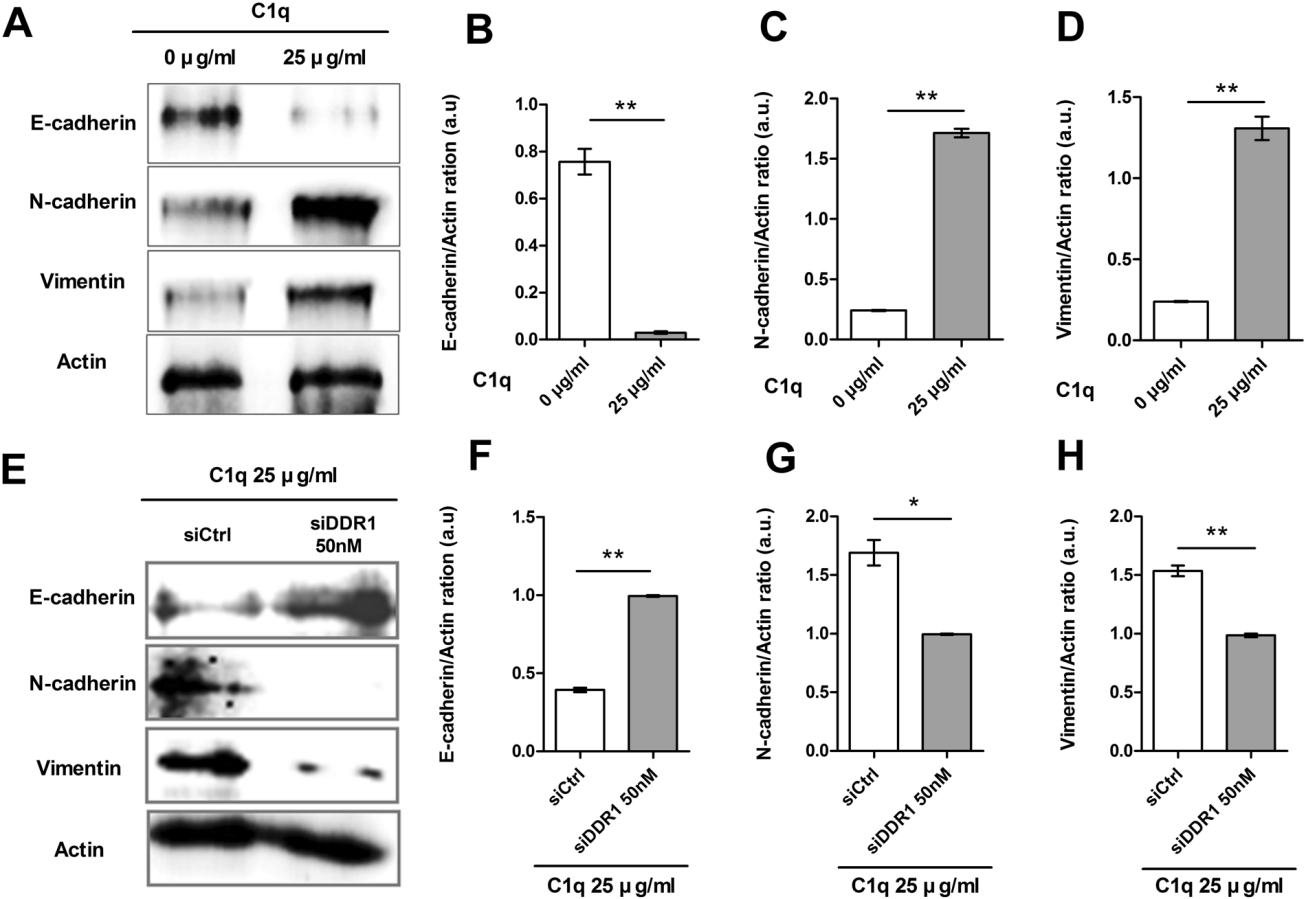
C1q regulated epithelial-mesenchymal transition (EMT) via DDR1 in HepG2 cells. The protein levels of the EMT markers E-cadherin, vimentin, and N-cadherin were assessed in cells treated with or without C1q by western blotting. (**A**) Cells were treated with the indicated doses of C1q for 18 h and E-cadherin, vimentin, N-cadherin, and actin expressions were analyzed by Western blot. (**B**) Bar graphs of E-cadherin/Actin, (**C**) N-cadherin/Actin, (**D**) Vimentin/Actin and ratios. (**E**) Cells were transfected with control (siCtrl) or siDDR1 for 36 h, treated with or without C1q for 6 h, and Western blotted for E-cadherin, vimentin, N-cadherin and actin. (**F**) Bar graphs of E-cadherin/Actin, (**G**) N-cadherin/Actin, (**H**) Vimentin/Actin and ratios. *P < 0.05, **P < 0.01.

EMT is a cellular program that allows immotile epithelial cells to convert to motile mesenchymal cells, and thereby, promotes the metastasis and invasion of tumor cells. EMT is also critical for the acquisition of malignant phenotypes by epithelial cancer cells^36,37^. Thus, we investigated the role of C1q in regulation of mesenchymal markers in cells. Our results showed that C1q enhanced the expressions of N-cadherin and vimentin and diminished that of E-cadherin in C1q treated HepG2 cells as compared with non-treated controls (Fig. 7A–D). Furthermore, C1q enhanced the expression of E-cadherin and diminished those of N-cadherin and vimentin in DDR1 siRNA-transfected HepG2 cells as compared with control siRNA-transfected cells (Fig. 7E–H), which suggested C1q promotes EMT and induced the migration and invasion of these cells.

### C1q bound to DDR1 in HepG2 cells

**Figure 8:**
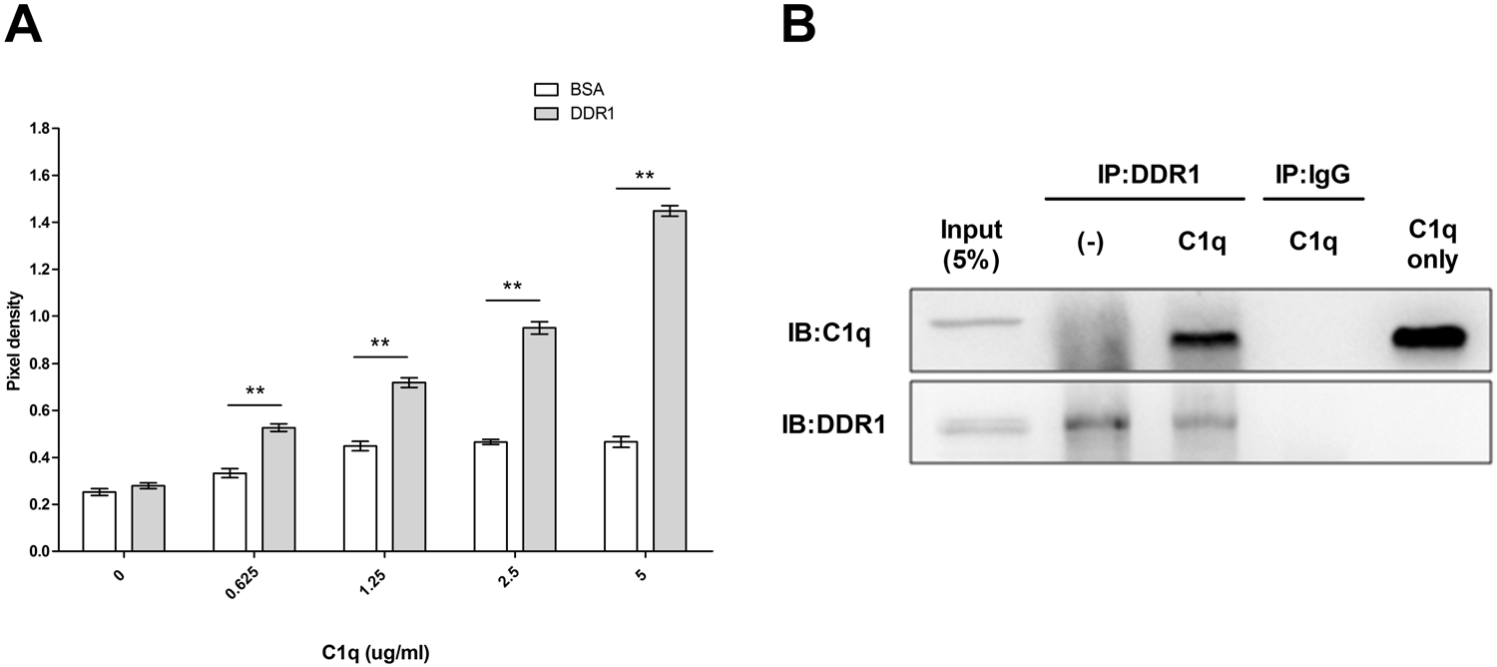
C1q bound directly to DDR1 in HepG2 cells. The interaction between C1q with DDR1 was investigated by ELISA and co-immunoprecipitation (Co-IP). (**A**) Binding of C1q with DDR1 by ELISA. Plates were coated with equal amounts of DDR1 (BSA was used as negative control) and incubated with diluted recombinant C1q. Absorbance was measured at 450 nm. C1q was found to dose-dependently bind to DDR1 with OD values higher than those of the control (BSA). (**B**) Co-immunoprecipitation of C1q and DDR1 examined by Western blotting. HepG2 cells were grown in 10 cm plates, treated with or without C1q for 1 h, lysed, and subjected to Co-IP. The lysates were with anti-DDR1 antibody or anti-IgG. The immunoprecipitates obtained were western blotted using anti-C1q or anti-DDR1 antibodies. As a protein input control, 5% of cell lysates were used. *P < 0.05, **P < 0.01.

In a recent study, it was reported DDR1 has high affinity for collagen, and that C1q has a collagen-like portion^10^. Based on these observations, we considered C1q binds directly to DDR1. We confirmed direct interactions between C1q and DDR1 using ELISA assay and Co-Immunoprecipitation (Co-IP). ELISA confirmed C1q directly and dose-dependently bound to DDR1 in HepG2 cells (Fig. 8A). And we used a Co-IP approach to detect the potentially direct binding of C1q and DDR1. A binding between C1q and DDR1 was detected in HepG2 cells (Fig. 8B).

## Discussion

During cancer development, the migration and invasion by cancer cells are associated with poor prognoses^26^. Thus, understanding the molecular pathways that orchestrate the tumorigenesis and metastasis of cancer cells is likely to aid the development of treatment strategies.

Hepatocytes are involved in the biosynthesis of plasma complement proteins, including C1q^38^, and it has been suggested hepatic complement proteins are involved in many liver functions, such as, glucose release, acute-phase response, inflammation, and immune complex clearance^38^. C1q is an innate immune molecule with a range of diverse ligands and functions^39^. However, the regulatory effects of C1q on tumor progression and the mechanisms responsible are largely unknown. Here, we report, for the first time, C1q binds directly to DDR1, and that this interaction induces the migration and invasion of HepG2 cells, and thus, presumably contributes to disease dissemination, which concurs with a previous reported that solid phase-bound C1q promotes fetal trophoblast cell migration and invasion^40^.

The up-regulations of MMP2 and MMP9 (downstream targets of DDR1) are considered to be important for the metastasis and invasion of HepG2 cells^26^. In this study, we found C1q significantly enhanced these expressions, and in-line with these results, it was found in a previous study that the expressional up-regulations of MMP2 and 9 modulate EMT and promote tumor cell migration and invasion^41^. These findings suggest C1q upregulates MMP2 and 9, and thus, enhances migration and invasion.

The MAPKs are a family of protein-serine/threonine kinases that are highly conserved in mammals. There are three major MAPKs, namely, p38, JNK, and ERK1/2^42^, and all three play critical roles in the development and progression of cancer^43^. Furthermore, it has been reported DDR1 interacts with MAPKs^15^. Accordingly, we examined the regulatory effects of C1q on MAPKs in HepG2 cells. C1q was found to activate MAPK signaling in these cells, and pharmacological blockades of individual MAPKs significantly attenuated the migratory phenotype. These observations concur with those of a previous study, in which MAPK inhibition abrogated the RANKL (receptor activator of nuclear factor kappa-B ligand) induced migration of breast cancer cells^44^. These findings suggest C1q modulates MAPK signaling via DDR1 to regulate the migratory and invasive phenotypes of cancer cells.

Activation of the PI3K/Akt pathway plays an important role in cancer tumorigenesis and progression^34,45^ and it has been reported PI3K and Akt are a downstream target of DDR1 activation^35^. In the present study, we found C1q activated PI3K/Akt signaling in HepG2 cells, and that pharmacological inhibition of the PI3K/Akt pathway significantly inhibited the ability of cells to migrate. Consistent with these results, it was previously reported inhibition of PI3K/Akt signaling inhibited the migration and invasion of colon cancer cells^45^.

To identify the molecule targeted by C1q during HCC progression, we treated HepG2 cells with or without C1q and evaluated the expressions of DDRs and of the well-known C1q receptors, gC1qR and cC1qR. Our results showed C1q significantly upregulated DDR1 expression, but not the expressions of gC1qR, cC1qR or DDR2. Furthermore, C1q was found to induce the DDR1 tyrosine phosphorylation. Several lines of evidence suggest that DDR1 is overexpressed in cancer cells and this upregulation is associated with aggressiveness^26,46^.

When DDR1 knocked down HepG2 cells were treated with or without C1q, knockdown was found to significantly attenuate C1q-induced migration and invasion, indicating C1q activates DDR1 to regulate cancer progression, which is in-line with previous reports that DDR1 plays important roles in the progression and metastasis of breast and lung cancer cells^32^.

EMT allows tumor cells to dissociate from tumor sites, and thereby, facilitates migration and invasion^47^ and it has been reported DDR1 signaling promotes EMT^48^. In the present study, C1q significantly reduced the expression of E-cadherin, but augmented the expressions of N-cadherin and vimentin (E-cadherin, N-cadherin, and vimentin are markers of EMT), which agrees with a previous report that DDR1 is involved in the collagen-induced upregulation of N-cadherin, and thus, promotes pancreatic tumor growth, invasion, and metastasis^48^. in addition, we examined the role of DDR1 in the C1q-mediated upregulations of MMP2 and 9. DDR1 gene knockdown resulted in significant reductions in the mRNA levels MMP2 and MMP9 in C1q-treated cells versus controls, suggesting DDR1 is a key regulator of the C1q-mediated aggressiveness of HepG2 cells.

The results of the present study support the notion that C1q directly activates DDR1 in liver cancer cells and enhances migratory and invasive phenotypes. Furthermore, our findings identify DDR1 as a target of C1q, and suggest that the interaction between the two promotes tumor aggressiveness.

## Methods

### Cell culture and reagents

HepG2 cells were obtained from the American Type Culture Collection (ATCC, Manassas, VA, USA) and cultured in Dulbecco’s modified Eagle’s medium (DMEM; Hyclone, Logan, UT, USA) containing 10% fetal bovine serum (FBS; Hyclone) and 1% of 100 units/ml of penicillin/streptomycin, and incubated at 37 °C in a humidified 5% CO_2_ incubator. Human C1q was obtained from Quidel (San Diego, CA, USA), Collagen I (endotoxin <1.0 EU/ml) from Advanced BioMatrix, Inc. (San Diego, CA, USA), serum free Opti-MEM from Invitrogen (Invitrogen, CA, USA), and WelFect-EX PLUS Transfection Reagent from Welgene (Welgene, Seoul, South Korea). Trizol reagent and the Superscript III kit were obtained from Invitrogen (Invitrogen, CA, USA). Radioimmunoprecipitation (RIPA) buffer, rabbit and mouse secondary antibodies, DDR1 antibody (sc-532), and anti-*β*-actin antibodies were obtained from Santa Cruz Biotechnology (Santa Cruz, CA). Total and/or phosphorylated-p38, -JNK, -ERK, -PI3K, -Akt, phosphor-tyrosine, and ERK antibodies were purchased from Cell Signaling. C1q antibody (ab71940) was obtained from Abcam (Cambridge, UK), and anti-Phospho-DDR1 (Y513) Antibody (P00905) from Bosterbio (California, USA). Control and DDR1 siRNA were from Santa Cruz Biotechnology (Santa Cruz, CA), and primers from Genotech (Daejeon, Korea). All other reagents were purchased from Sigma-Aldrich (St. Louis, MO, USA).

### siRNA transfection

HepG2 cells were transfected with human DDR1 specific siRNA or control siRNA for 36 h using WelFect-EX PLUS Transfection Reagent (Welgene, Seoul, South Korea), according to the manufacturer’s instructions. Briefly, HepG2 cells were cultured in 24-well plates in growth medium for 18 h. Then remove the growth medium and wash with serum free medium once. Add 0.3 ml serum free medium and incubate into CO_2_ incubator until adding DNA complex. Cells were then incubated with transfection complex containing 0.5 μg human DDR1 specific siRNA, 0.5 μg control siRNA, and transfection reagent (2 μg Welfect-Ex and 1.5 μg Enhancer-Q) in serum free Opti-MEM (Invitrogen, USA). After incubate during 3 h, add complete growth medium. Transfection was continued for 36 h. siRNA transduction efficacies were confirmed by Western blotting.

### Cell migration assay

Cell migration was measured using a monolayer scratch injury assay. Briefly, HepG2 cells were cultured in 48-well plates for 18 h. Then a uniform scratch (about 500 µm) was gently performed in the monolayers using a sterile pipette tip (200 µl). Cells were then washed gently with phosphate-buffered saline (PBS) to remove cellular debris to ensure acellular lines. To initiate migration, cells were treated with or without C1q in serum-free medium. Cells were then allowed to migrate for 18 h, and images of migration under static culture in an incubator containing 5 per cent CO_2_ at 37 °C without serum were acquired at 0 and 18 h. Wound widths were measured using an Olympus IX71 microscope (Olympus Optical Co. Ltd., Tokyo, Japan). Selected representative images were obtained, and results were quantified using Image J software (NIH, Bethesda MD, USA).

### Cell matrigel invasion assay

Matrigel invasion assays were performed using 6-well transwell plates (Becton Dickinson, Bedford, MA), as previously described^25^. HepG2 cells were cultured in medium with or without C1q, and seeded in top chambers with a Matrigel-coated membrane (6-well insert; pore size, 8 mm). After 18 hours of culture, invasive cells moved through the matrix and adhered to the bottom membrane of the insert. Cells that did not invade through pores were removed, and invading cells were stained with 0.1% crystal violet. Cells were photographed as described above for the migration assay, and counted in at least 3 fields (counts were then averaged).

### Flow cytometry

The expressions of cell surface antigens of DDR1 were evaluated by using FACS Calibur (Beckton-Dickinson, San Diego, CA). Cells were cultured in 12-well cell culture plates and incubated in a 5% CO_2_ with humidified air at 37 °C. Cells were serum starved for 2 h before complement treatment. C1q was diluted to the desired concentration with culture medium and added to each well. Cell culture was continued for 18 h at 37 °C in 5% CO_2_ and then 1 × 10^5^ cells were collected, centrifuged at 1200 rpm for 5 min, and re-suspended in 100 μL of 0.1% sodium azide buffer. Anti- DDR1 (1:1000 dilution) primary antibody (1 μg) was then added and cells were incubated at room temperature in the dark for 30 min, washed twice and 1 μg FITC-conjugated secondary antibody (1:1000 dilution) in 0.1% sodium azide was added. Cells were then incubated for 30 min in the dark at room temperature, washed twice, treated with 400 μL 0.1% sodium azide buffer was added, and subjected to flow cytometry.

### Real-time PCR

Total RNA was extracted using Trizol reagent, according to the manufacturer’s instructions. Total RNA (2 µg) was used to synthesize cDNA using a Super Script TM III kit. mRNA expressions were quantitatively using the ABI Real-Time PCR system (Applied Biosystems, Inc., Forster City, CA) and SYBR Green PCR Master Mix (Life Technologies). GAPDH was used as the internal control. The primer sequences used for real-time PCR are shown in Table 1.

**Table 1:** Primers used for PCR.

Gene	Forward	Reverse	Size
DDR1	GCTGGGGTCAGGAGGTGAT	AATACATTGTCTGCC	207
DDR2	GAGGCCAGATTCCAGATGAG	GGTGTGCAAGTCAATCTGCA	167
gC1qR	ACAACAGCATCCCACCAA	GCCTTCTTGCCATCAT	136
cC1qR	AAGGTTTGCAGACAAGCCAG	CCTTCTTGGTGCCAGG	240
Beta1	AGAATCCAGAGTGTCCCACTG	TTCCCTCATACTTCGGATTGA	237
Cd91	TCTGCGATGGCGACAATGAC	AATGGAGCAATGGAA	212
Cd93	TCCTGTGCAAGGAGAAGGCC	AGGCACAGGTCACC	192
LAIR-1	CAGCATCCAGAAGGTTCGTTA	GAACAGGCAGGTGAC	176
GAPDH	GTTAGGAAAGCCTGCCGGTG	GCATCACCCGGAGGAGAAATC	118

### Immunoprecipitation and Western blot analysis

Cells were lysed in ice-cold RIPA buffer for 40 min and centrifuged (12,000 *g*) for 20 min at 4 *°*C. Protein concentrations were measured using the bicinchoninic acid method. For immunoprecipitation, total cell lysates were incubated with Protein A agarose (Santa Cruz, CA) with an rabbit polyclonal DDR1 antibody. Bound proteins were eluted with 2 × SDS-sample buffer and analyzed by Western blotting. Briefly, 20 μg of lysates were subjected to SDS-polyacrylamide gel electrophoresis and transferred to polyvinylidene difluoride (PVDF) membranes (Amersham Pharmacia Biotech). Membranes were blocked with 5% bovine serum albumin (BSA) in Tris-buffered saline containing 0.1% Tween-20 (TBST) for 1 h at room temperature, probed with primary antibodies (as indicated) at 4*°*C overnight, washed with TBST 4 times, and incubated with horseradish peroxidase (HRP)-conjugated secondary antibody for 45 min at room temperature. Membranes were then rewashed 3 times with TBST and proteins were visualized using an enhanced chemiluminescence detection kit (Millipore, MA, USA). Membranes were measured using Fusion Fx gel documentation system (Vilber Lourmat, Marne-la-Vallee, France). Western band densitometry calculations were perforned using Gel Quant NET software.

### ELISA

C1q binding was investigated by ELISA. Briefly, 96-well plates were coated with 2 µg of purified full length human DDR1 protein (Quidel, San Diego, CA, USA) in 200 μl of carbonate buffer at 4 *°*C overnight. BSA was included as a negative control. Plates were blocked with 10% FBS in PBS and then increasing concentrations of C1q (0, 0.625, 1.25, 2.5, or 5 µg/mL) were added. Protein binding was assessed using anti-C1q (dilution 1:100) and subsequent incubation with HRP-conjugated anti-rabbit IgG (Sigma) (1:500), both incubations were performed at 37 °C for 2 hours. Wells were then washed 3 times, and 100 μl of TMB solution was added immediately to each well, and incubated for 15 minutes at room temperature. After adding 50 μl of 2 N sulfuric acid (Stop Solution) to each well, absorbance at 450 nm was measured using a microplate reader Multiskan Spectrum (Thermo Scientific).

### Statistical analysis

All values are expressed as means ± S.D. Statistical significance was determined using the Student’s *t*-test. Statistical significance was accepted for p values < 0.05.

## Electronic supplementary material

Supplementary Information

## References

[1] Alves RC (2011). Advanced hepatocellular carcinoma. Review of targeted molecular drugs. Annals of hepatology..

[2] Waly RS, Yangde Z, Yuxiang C (2012). Hepatocellular carcinoma: focus on different aspects of management. ISRN Oncology..

[3] Giannini EG (2015). Prognosis of untreated hepatocellular carcinoma. Hepatology..

[4] Tang H, Li RP, Liang P, Zhou YL, Wang GW (2015). miR-125a inhibits the migration and invasion of liver cancer cells via suppression of the PI3K/AKT/mTOR signaling pathway. Oncology letters..

[5] Roba MT (2007). Serum Levels of Complement C1Q, C3 and C4 in Patients at Different Stages of Chronic Hepatitis C ViralInfection. World Journal of Medical..

[6] Petry F (2001). Reconstitution of the complement function in C1q-deficient (C1qa^−/−^) mice with wild-type bone marrow cells. The Journal of immunology..

[7] Bulla R (2016). C1q acts in the tumour microenvironment as a cancer-promoting factor independently of complement activation. Nature communications..

[8] Araki K (1982). Serum C1q levels in patients with various liver disease. Kanzo..

[9] Yoshida H (1983). C1q and immune complexes in liver cirrhosis sera. Tohoku Journal Of Experimental Medicine..

[10] Reid KB (1974). A collagen-like amino acid sequence in a polypeptide chain of human C1q (a subcomponent of the first component of complement). The Biochemical journal..

[11] Eggleton P, Reid KB, Tenner AJ (1998). C1q—how many functions? How many receptors?. Trends in cell biology..

[12] Nayak A, Pednekar L, Reid KB, Kishore U (2012). Complement and non-complement activating functions of C1q: A prototypical innate immune molecule. Innate Immunity..

[13] Lu JH (2008). The classical and regulatory functions of C1q in immunity and autoimmunity. Cellular and molecular Immunology..

[14] Son, M., Santiago-Schwarz, F., Al-Abed, Y. & Diamond, B. C1q limits dendritic cell differentiation and activation by engaging LAIR-1. *Proceeding of the National Academy of Sciences of the United States of America*. **109**, 3160-3167 (2012). 10.1073/pnas.1212753109 PMC3503216 23093673

[15] Vogel WF, Abdulhussein R, Ford CE (2006). Sensing extracellular matrix: an update on discoidin domain receptor function. Cellular signaling..

[16] Shrivastava A (1997). An orphan receptor tyrosine kinase family whose members serve as non integrin collagen receptors. Molecular Cell..

[17] Vogel W, Gish GD, Alves F, Pawson T (1997). The discoidin domain receptor tyrosine kinases are activated by collagen. Molecular Cell..

[18] Leitinger B, Kwan AP (2006). The discoidin domain receptor DDR2 is a receptor for type X collagen. Matrix Biology..

[19] Xu H (2011). Collagen binding specificity of the discoidin domain receptors: Binding sites on collagens II and III and molecular determinants for collagen IV recognition by DDR1. Matrix Biology..

[20] Li Y, Lu X, Ren X, Ding K (2015). Small Molecule Discoidin Domain Receptor Kinase Inhibitors and Potential Medical Applications. Journal of Medicinal Chemistry..

[21] Iwai L, Luczynski M, Huang P (2014). Discoidin domain receptors: a proteomic portrait. Cellular and Molecular Life Sciences..

[22] Diaz, I. B., Wang, J., Mort, J. S. & Komarova, S. V. Collagen Type I as a Ligand for Receptor-Mediated Signaling. *Frontiers in Physics*. **5**, 10.3389/fphy.2017.00012 (2017).

[23] Carafoli F, Hohenester E (2013). Collagen recognition and transmembrane signalling by discoidin domain receptors. Biochimica Et Biophysica Acta..

[24] Fu HL (2013). Discoidin domain receptors: unique receptor tyrosine kinases in collagen-mediated signaling. Journal of Biological Chemistry..

[25] Poudel B, Lee YM, Kim DK (2015). DDR2 inhibition reduces migration and invasion of murine metastatic melanoma cells by suppressing MMP2/9 expression through ERK/NF-κB pathway. Acta. Biochimica et biophysica Sinica..

[26] Park HS (2007). Overexpression of discoidin domain receptor 1 increases the migration and invasion of hepatocellular carcinoma cells in association with matrix metalloproteinase. Oncology reports..

[27] Rudra-Ganguly N (2014). Discoidin domain receptor 1 contributes to tumorigenesis through modulation of TGFBI expression. PLoS One..

[28] Leitinger B (2014). Discoidin Domain Receptor Functions in Physiological and Pathological Conditions. International Review of Cell and Molecular Biology..

[29] Petry F, Reid KB, Loos M (1989). Molecular cloning and characterization of the complementary DNA coding for the B-chain of murine Clq. FEBS Letters..

[30] Muragaki Y (1991). The alpha 2(VIII) collagen gene. A novel member of the short chain collagen family located on the human chromosome 1. The Journal of Biological Chemistry..

[31] Zutter MM, Edelson BT (2007). The α2β1 integrin: A novel collectin/C1q receptor. Immunobiology..

[32] Multhaupt HA, Leitinger B, Gullberg D, Couchman JR (2106). Extracellular matrix component signaling in cancer. Advanced drug delivery reviews..

[33] Li NY (2013). An MAPK-dependent pathway induces epithelial-mesenchymal transition via Twist activation in human breast cancer cell lines. Surgery..

[34] Cai Y (2014). Inhibition of PI3K/Akt/mTOR signaling pathway enhances the sensitivity of the SKOV3/DDP ovarian cancer cell line to cisplatin *in vitro*. Chinese journal of cancer research..

[35] Ongusaha PP (2013). p53 induction and activation of DDR1 kinase counteract p53-mediated apoptosis and influence p53 regulation through a positive feedback loop. The EMBO Journal..

[36] Chang CJ (2011). p53 regulates epithelial-mesenchymal transition and stem cell properties through modulating miRNAs. Nature cell biology..

[37] Yang J, Weinberg RA (2008). Epithelial-mesenchymal transition: at the crossroads of development and tumor metastasis. Developmental Cell..

[38] Phieler J, Garcia Martin R, Lambris JD, Chavakis T (2013). The role of the complement system in metabolic organs and metabolic diseases. Seminars in immunology..

[39] Kouser L (2015). Emerging and novel functions of complement protein C1q. Frontiers in immunology..

[40] Agostinis C (2010). An alternative role of C1q in cell migration and tissue remodeling: contribution to trophoblast invasion and placental development. The Journal of immunology..

[41] Epanchintsev A, Shyamsunder P, Verma RS, Lyakhovich A (2015). IL-6, IL-8, MMP-2, MMP-9 are overexpressed in Fanconi anemia cells through a NF-κB/TNF-α dependent mechanism. Molecular carcinogenesis..

[42] Liu FY (2014). CCR7 regulates cell migration and invasion through MAPKs in metastatic squamous cell carcinoma of head and neck. International journal of oncology..

[43] Dhillon AS, Hagan S, Rath O, Kolch W (2007). MAP kinase signalling pathways in cancer. Oncogene..

[44] Tang ZN (2011). RANKL-induced migration of MDA-MB-231 human breast cancer cells via Src and MAPK activation. Oncology reports..

[45] Zhang X (2015). miR-218 inhibits the invasion and migration of colon cancer cells by targeting PI3K/Akt/mTOR signaling pathway. International Journal of molecular medicine..

[46] Valiathan RR (2012). Discoidin domain receptor tyrosine kinases: new players in cancer progression. Cancer Metastasis Reviews..

[47] Koh MR (2015). Discoidin domain receptor 1 is a novel transcriptional target of ZEB1 in breast epithelial cells undergoing H-Ras-induced epithelial to mesenchymal transition. International journal of cancer..

[48] Shintani Y (2008). Collagen I–mediated up-regulation of N-cadherin requires cooperative signals from integrins and discoidin domain receptor 1. The journal of cell biology..

