# Supplementary material for: Complement C1q stimulates the progression of hepatocellular tumor through the activation of discoidin domain receptor 1
Source: Sci Rep. 2018 Mar 20;8:4908. doi: 10.1038/s41598-018-23240-6 (PMC5861131; doi:10.1038/s41598-018-23240-6)

Complement C1q stimulates the progression of hepatocellular tumor through the activation of discoidin domain receptor 1

Ji-Hyun Lee[1*], Barun Poudel[1*], Hyeon-Hui Ki[1], Sarmila Nepali[1], Young-Mi Lee[2], Jeon-Soo Shin[3], Dae-Ki Kim[1#]

[1]Department of Immunology and Institute for Medical Sciences, Chonbuk National University Medical School, Jeonju, Jeollabuk-do 54907, Republic of Korea.

[2]Department of Oriental Pharmacy, College of Pharmacy and Wonkwang-Oriental Medicines Research Institute, Wonkwang University, Iksan, Jeollabuk-do 54538, Republic of Korea.

[3]Department of Microbiology, BK21 PLUS for Medical Sciences, Yonsei University College of Medicine, Seoul 03722, Republic of Korea.

Supplementary Information 1

Figure S1.

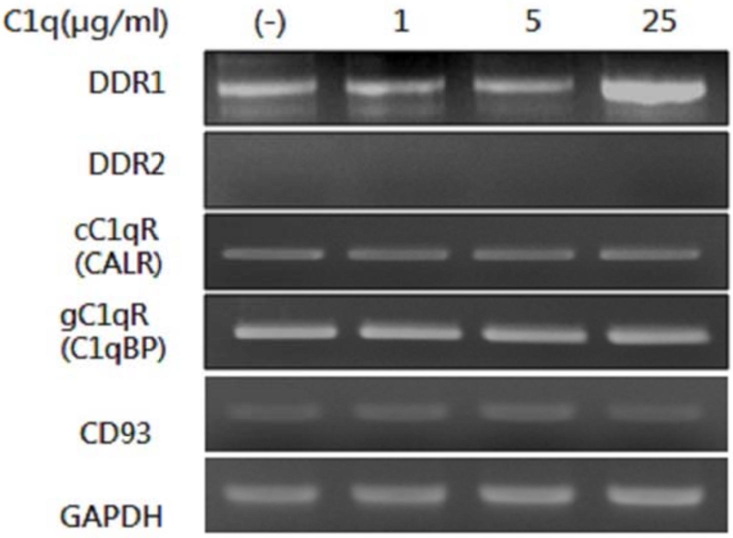

Cells treated with or without C1q were harvested and the mRNA levels of DDR1, DDR2, cC1qR, gC1qR and CD93 were assessed by RT-PCR. As a result, the enhanced expression of DDR1 was observed in C1q-treated cells. However, DDR2 was not expressed in HepG2 cells. And the expression of C1qBP (gC1qR), calreticulin (cC1qR), and CD93 were expressed but the expression levels were not increased by the stimulation of C1q.

Supplementary Information 2

Additional experiments were performed using SUN182 cells to determine whether the same results were obtained with other HCC cell lines. As a result, the results are similar to the cell HepG2 who had used in the experiment.

Figure S2.

SNU182 Cells treated with or without C1q were harvested and the mRNA levels of DDR1 by RT-PCR. (A) Bars represent the relative band intensity levels of DDR1. (B) Cells were treated with the indicated doses of C1q for 18 h and DDR1 expressions were analyzed by Western blot. **P*<0.05, ** *P*<0.01.

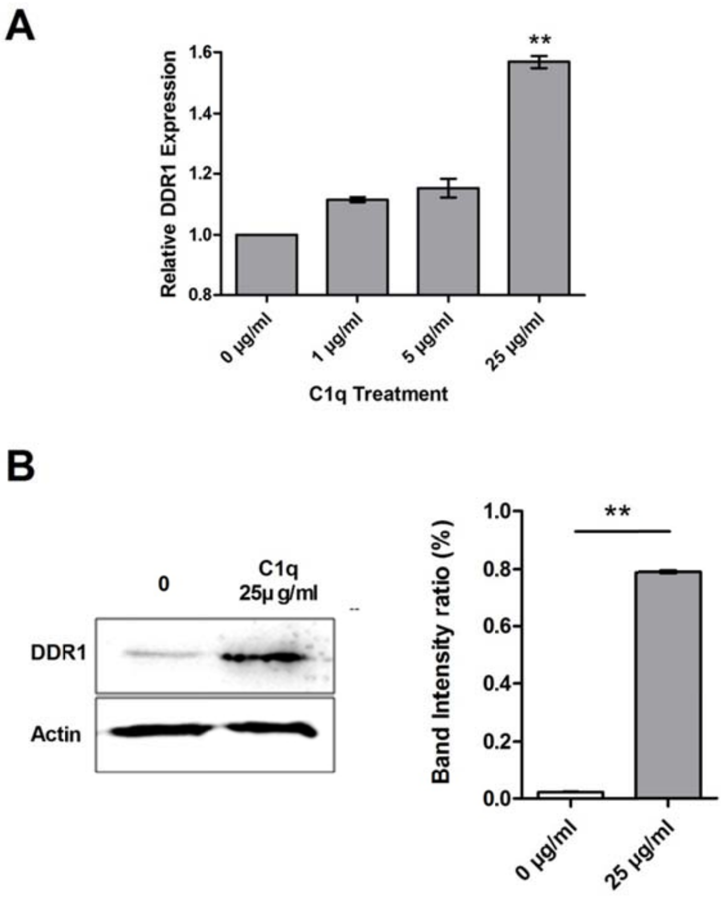

We gained the western blot image using by Fusion Fx gel documentation system (Vilber Lourmat, Marne-la-Vallee, France) then captured using image capture program. Finally the western band densitometry calculations were done using Gel Quant NET software. In order to minimize the measurement error in the software Gel QuantNet, the background brightness has been moderately adjusted.

Supplementary Information total gel

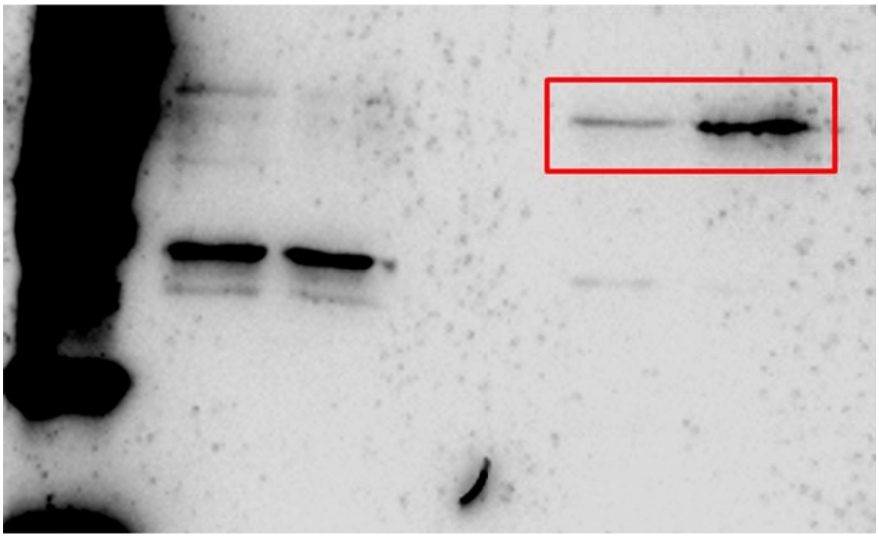

Supplement: Supplementary file 1 — Supplementary Information [file 41598_2018_23240_MOESM1_ESM.pdf]
